# Diagnostic and prognostic potential of serum microRNA-4651 for patients with hepatocellular carcinoma related to aflatoxin B1

**DOI:** 10.18632/oncotarget.16027

**Published:** 2017-03-08

**Authors:** Xue-Min Wu, Zhi-Feng Xi, Pinhu Liao, Hong-Dong Huang, Xiao-Ying Huang, Chao Wang, Yun Ma, Qiang Xia, Jin-Guang Yao, Xi-Dai Long

**Affiliations:** ^1^ Department of Pathology, The Affiliated Hospital of Youjiang Medical University for Nationalities, Baise, China; ^2^ Department of Liver Surgery, Ren Ji Hospital, School of Medicine, Shanghai Jiao Tong University, Shanghai, China; ^3^ Department of Medicine, The Affiliated Hospital of Youjiang Medical University for Nationalities, Baise, China; ^4^ Division of Medicine, Beijing Shijitan Hospital, Capital Medical University, Beijing, China; ^5^ Department of Pathology, The Affiliated Tumor Hospital, Guangxi Medical University, Nanning, China

**Keywords:** microRNA-4651, aflatoxin B1, hepatocellular carcinoma, diagnostic and prognostic biomarkers

## Abstract

**Background:**

The serum microRNAs have been reported as potential biomarkers for hepatitis virus-related hepatocellular carcinoma (HCC); however, their role in aflatoxin B1 (AFB1)-related HCC to has not yet been evaluated.

**Materials and Methods:**

We conducted a case-control study, including 366 HCC cases and 662 controls without any evidence of tumors, to identify and assess diagnostic and prognostic potential of serum microRNAs for AFB1-related HCC. The sensitivity, specificity, and area under the receiver operating characteristic curve (AUC) were used to elucidate diagnostic performance, and to compare the microRNAs with α-fetoprotein (AFP) at a cutoff of 20 ng/mL (AFP20) and 400 ng/mL (AFP400).

**Results:**

We found 8 differentially expressed microRNAs via the microRNA array analysis; however, only microRNA-4651 was further identified to detect AFB1-positive HCC but not AFB1-negative HCC. For AFB1-positive HCC, microRNA-4651 showed higher accuracy and sensitivity than AFP400 (AUC, 0.85 *vs*. 0.72; Sensitivity, 78.1% vs. 43.0%). Compared to AFP20, microRNA-4651 exhibited higher potential in identifying small-size (0.68 vs. 0.84 for AUC and 36.7% *vs*. 75.5% for sensitivity, respectively) and early-stage HCC (0.69 *vs*. 0.84 for AUC and 38.7% *vs*. 75.7% for sensitivity, respectively). Additionally, miR-4651 was also associated with HCC prognosis (hazard risk value, 2.67 for overall survival and 3.62 for tumor recurrence analysis).

**Conclusions:**

These data suggest that serum microRNA-4651 may be a useful marker for HCC diagnosis and prognosis, especially AFB1-positive cases.

## INTRODUCTION

Hepatocellular carcinoma (HCC), accounting for more than 90% of primary liver cancer, is the sixth most common malignant tumor, and the third-leading cause of cancer-related death in the world.[1] From a global perspective, the infection of hepatitis virus B (HBV) or C (HCV) and the exposure to aflatoxin B1 (AFB1) are the two main risk factors for HCC, especially in the highly epidemic areas of many Asian and African countries including China [1]. With early detection, HCC patients often have a good chance of a successful curative operation, and the 5-year overall survival (OS) rate is more than 50%. However, with late diagnosis, 5-year OS rate is reduced to less than 10%, and the dismal prognosis is largely caused by late detection of the tumors after disease progression [1–3]. Therefore, early detection of HCC at a curative stage offers the best chance of survival for these patients.

Currently, the early diagnosis of HCC is based on imaging examination and serological tests. Although advances in computed tomography (CT) and magnetic resonance imaging (MRI) technology have greatly improved the early screening of HCC, these procedures are costly and not suitable for daily clinical practice [3, 4]. For serological tests, α-fetoprotein (AFP) is the most widely used biomarker for HCC worldwide. However, the accuracy of AFP is modest (with sensitivity of 40 - 65%; and specificity of 87 - 96%), and about 30% of cases of early-stage HCC are missed using AFP analysis. Additionally, serum AFP levels of benign liver diseases, including hepatitis and cirrhosis, may give false-positive results [3]. Thus, performance of this biomarker to detect disease can be inadequate.

Increasing evidence has shown a link between aberrant expression of microRNAs and HCC [5–7]. In particular, microRNAs are highly stable in circulation and expression patterns seem to be tissue-specific, suggesting circulating microRNAs may be potentially ideal biomarkers for early-stage HCC [7–9]. Recent studies have demonstrated that serum microRNAs can identify preclinical HCC related to hepatitis viruses such as HBV and HCV [10–20]. However, information on whether serum microRNAs are correlated with AFB1-related HCC is limited. Therefore, monitoring circulating microRNA signatures during AFB1 exposure and correlated diseases may be clinically relevant as a non-invasive diagnostic tool for early detection of HCC. In this study, we assessed the significance of the serum microRNAs in detecting HCC from individuals with positive AFB1 exposure. Our findings revealed that microRNA-4651 (miR-4651) could serve as a potential biomarker for early diagnosis of AFB1-related HCC.

## RESULTS

### The characteristics of HCC cases and controls

According to eligibility criteria listed in Table 1, we collected 1028 serum samples from the five groups of participants (Table 2). For each group, the age, sex, race, and the time and location sample collection were well-matched. About 40% of HCC cases were in the early stage of the BCLC system.

**Table 1:** Eligibility criteria for the recruited participants

Group	Criteria
Healthy control without positive AFB1 (Group 1)	1. Negative HBV markers (HBsAg, HBeAg, anti-HBe, anti-HBc, and HBV DNA)
	2. Negative anti-HCV
	3. No liver diseases and other systematic diseases
	4. Persistently normal AST and ALT levels
	5. Negative ln(AAA)
Healthy control with positive AFB1 (Group 2)	1. Negative HBV markers (HBsAg, HBeAg, anti-HBe, anti-HBc, and HBV DNA)
	2. Negative anti-HCV
	3. No liver diseases and other systematic diseases
	4. Persistently normal AST and ALT levels
	5. Positive ln(AAA)
Control with liver diseases exception tumors (Group 3)	1. Negative HBV markers (HBsAg, HBeAg, anti-HBe, anti-HBc, and HBV DNA)
	2. Negative anti-HCV
	3. Without evidence of liver tumors
	4. Persistently or intermittent elevation in AST or ALT levels
	5. Positive ln(AAA)
AFB1-related HCC (Group 4)	1. Diagnosis based on hepatic ultrasound together with CT and/or MRI by at least two imaging technologists
	2. Confirmed histopathologically by two independent pathologists
	3. Negative HBV markers (HBsAg, HBeAg, anti-HBe, anti-HBc, and HBV DNA)
	4. Negative anti-HCV
	5. Positive ln(AAA)
	6. No pre-operative chemotherapy, radiotherapy, transarterial chemoembolization or ablation before collection of blood samples
Non-AFB1-related HCC (Group 5)	1. Diagnosis based on hepatic ultrasound together with CT and/or MRI by at least two imaging technologists
	2. Confirmed histopathologically by two independent pathologists
	3. Negative HBV markers (HBsAg, HBeAg, anti-HBe, anti-HBc, and HBV DNA)
	4. Negative anti-HCV
	5. Negative ln(AAA)
	6. No pre-operative chemotherapy, radiotherapy, transarterial chemoembolization or ablation before collection of blood samples

Abbreviation. HBV, hepatitis B virus; HBsAg, HBV surface antigen; HBeAg, HBV e antigen; anti-HBe, antibody against HBeAg; anti-HBc, HBV core antibody; anti-HCV, antibody against hepatitis C virus; AST, aspartate aminotransferase; ALT, alanine aminotransferase; AFB1, aflatoxin B1; ln (AAA), the logarithmical value of serum AFB1 albumin adducts levels of peripheral blood; HCC, hepatocellular carcinoma.

**Table 2:** Clinic-pathological characteristics of study subjects

Variable	Group 1 (*n*= 338)	Group 2 (*n*= 292)	Group 3 (*n*= 32)	Group 4 (*n*= 279)	Group 5 (*n*= 87)	*p*-value
Age, mean±SD (yrs)	49.4±10.9	50.2±10.4	50.9±6.9	49.9±11.22	50.2±11.9	Matched
Gender (Female), *n* (%)	102 (30.2)	90 (30.8)	10 (31.3)	86 (30.8)	27 (31.0)	Matched
Race (Han), *n* (%)	176 (52.1)	155 (53.1)	17 (53.1)	148 (53.0)	46 (52.9)	Matched
Race (Zhuang), *n* (%)	162 (47.9)	137 (46.9)	15 (46.9)	131 (47.0)	41 (47.1)	Matched
AST (≤45 U/L), *n* (%)	338 (100.0)	292 (100.0)	22 (68.7)	118 (42.3)	34 (39.1)	<0.001/0.60*
ALT (≤45 U/L), *n* (%)	338 (100.0)	292 (100.0)	21 (65.6)	114 (40.9)	32 (36.8)	<0.001/0.50*
AFP (≤20 ng/mL), *n* (%)	338 (100.0)	292 (100.0)	24 (75.0)	108 (38.7)	33 (37.9)	<0.001/0.90*
AFP (≤400 ng/mL), *n* (%)	338 (100.0)	292 (100.0)	32 (100.0)	159 (57.0)	48 (55.2)	<0.001/0.20*
Cirrhosis, *n* (%)	0 (0.0)	0 (0.0)	27 (84.4)	203 (72.8)	64 (73.6)	<0.001/0.88*
Tumor size (≤3 cm), *n* (%)	-	-	-	98 (35.1)	25 (28.7)	0.64
BCLC stage						0.96
0-A, *n* (%)	-	-	-	111 (39.8)	35 (40.2)	-
B, *n* (%)	-	-	-	91 (32.6)	27 (31.1)	-
C, *n* (%)	-	-	-	77 (27.6)	25 (28.7)	-

*The *p* value indicates the statistical significance for the differences among Group 1, 2, 3, and 4/between Group 4 and 5. Abbreviations: AST, aspartate transaminase; ALT, alanine transaminase; AFP, α-fetoprotein; BCLC stage, the Barcelona Clinic Liver Cancer staging system.

### Differential expression of serum microRNAs levels between AFB1-positive HCC cases and controls

**Figure 1:**
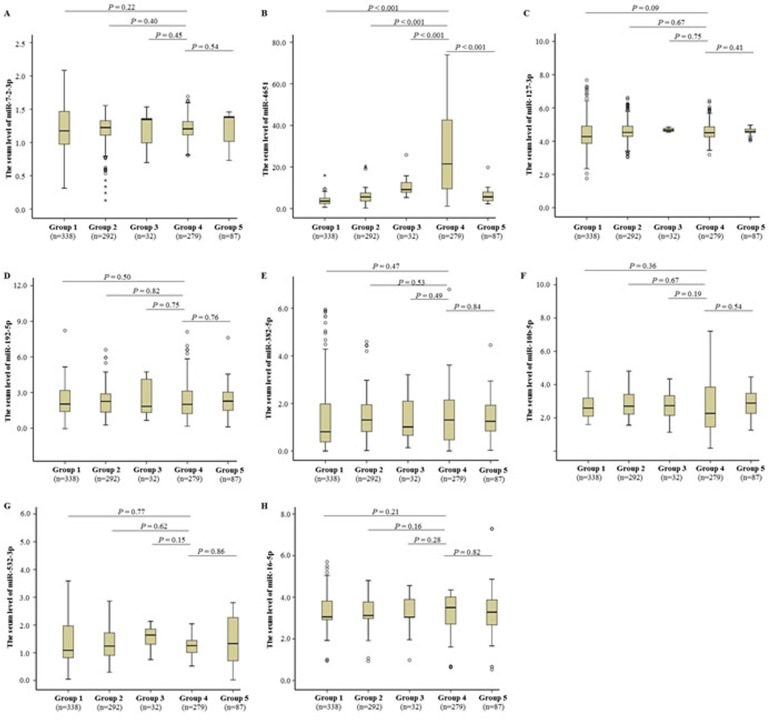
Differentially expressed microRNAs in the serum samples Eight differentially expressed microRNAs in the discovery, including miR-7-2-3p **A.**, miR-4651 **B.**, miR-127-3p **C.**, miR-192-5p **D.**, miR-382-5p **E.**, miR-10b-5p **F.**, miR-532-3p **G.**, and miR-16-5p **H.**, were further analyzed using TaqMan-PCR method in the training stage. The relative levels of microRNA expression were calculated according to 2^−ΔCT^ method (*see Materials and Methods*). The microRNA data are shown as box plots, with horizontal lines representing the median, the bottom and the top of the boxes representing the 25th and 75th percentiles, respectively. We compared expression data between groups using the Mann-Whitney *U* test.

In this study, we first examined the serum microRNA profiles in AFB1-positive HCC cases (randomly selected from Group 4, *n* = 6) compared to healthy controls with positive AFB1 (randomly selected from Group 2, *n* = 6) using RT^2^ Profiler PCR Array, and identified 8 significantly different microRNAs (including miR-7-2-3p, miR-4651, miR-127-3p, miR-192-5p, miR-382-5p, miR-10b-5p, miR-532-3p, and miR-16-5p) between cases and controls (Table 3). Next, we further investigated the serum expression profiles of these 8 microRNAs in all participants using TaqMan-PCR technique (Figure 1). Mann-Whitney *U*-test showed that only miR-4651 was increased in AFB1-positive HCC patients compared to controls (*P* < 0.001). Furthermore, AFB1-positive HCC cases featured higher serum miR-4651 level than AFB1-negative patients (*P* < 0.001), suggesting this microRNA might be specific for AFB1-positive HCC.

**Table 3:** Eight candidate microRNAs displaying higher levels in HCC cases with positive AFB1 than controls with positive AFB1 in the discovery cohort

	miRNA	Average Ct value		CV		Fold Change
No.		Controls	Cases	Controls	Cases	
01	miR-7-2-3p	28.62	22.03	0.0171	0.0236	10.71
02	miR-4651	28.12	12.96	0.0238	0.0694	4071.41
03	miR-127-3p	26.16	21.62	0.0317	0.0167	10.31
04	miR-192-5p	28.99	17.57	0.0328	0.0102	302.61
05	miR-382-5p	28.91	21.91	0.0090	0.0050	14.19
06	miR-10b-5p	28.77	17.77	0.0115	0.0523	227.75
07	miR-532-3p	26.66	22.83	0.0154	0.0197	3.57
08	miR-16-5p	25.67	20.32	0.0105	0.0305	4.54

Abbreviations: HCC, hepatocellular carcinoma, AFB1, aflatoxin B1; CV, coefficient of variation.

### Diagnostic performance of serum miR-4651

**Figure 2:**
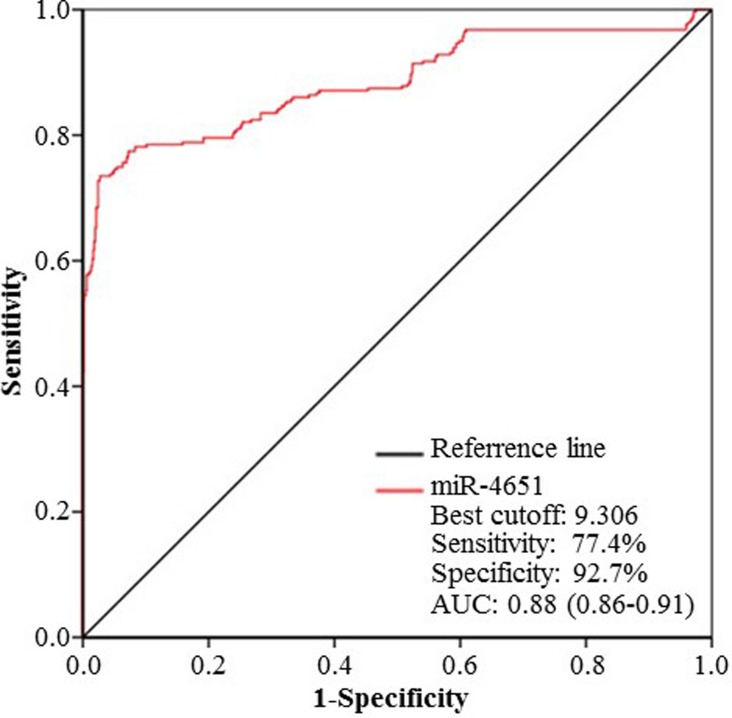
The receiver operating characteristic (ROC) curve of serum miR-4651 for discrimination To obtain the best-off value of miR-4651 for discrimination, ROC curve was constructed using miR-4651 expression data (2^−ΔCT^) of Group 1, 2, 3, and 4 in the training stage. *Abbreviations*: AUC, the area under the ROC curve.

**Figure 3:**
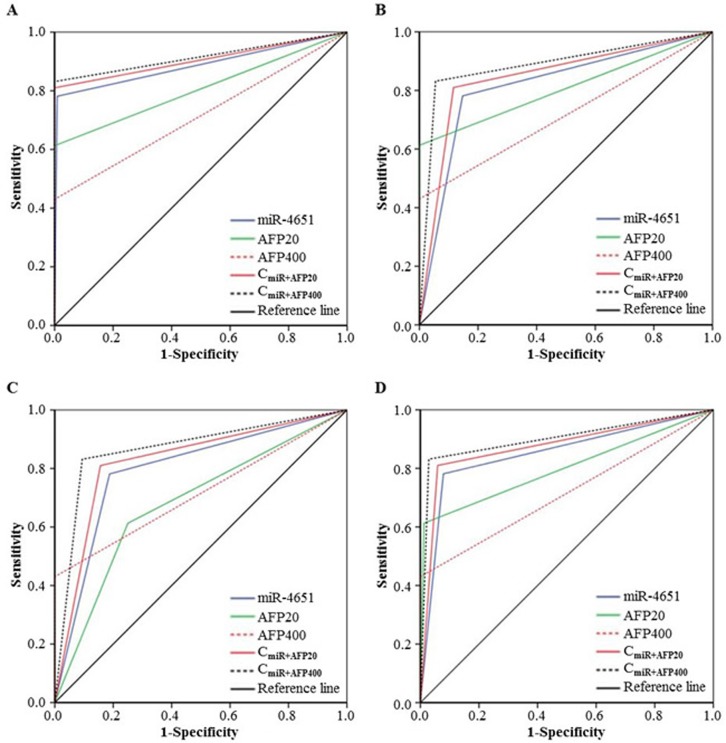
Comparison of receiver operating characteristic curve (ROC) for miR-4651 and AFP to detect aflatoxin B1 (AFB1)-positive hepatocellular carcinoma ROC was used to differentiate patients with AFB1-positive HCC (Group 4, *n* = 279) from healthy controls without positive AFB1 exposure (Group 1, *n* = 338) **A.**, from healthy controls with positive AFB1 (Group 2, *n* = 292) **B.**, from control with liver diseases exception tumors (Group 3, *n* = 32) **C.**, or the combination of Group 1, 2, and 3 (*n* = 662) **D.** *Abbreviations*: AFP20, the cutoff of 20 ng/mL AFP; AFP400, the cutoff of 400 ng/mL AFP; C_miR+AFP20_, the combination of miR-4651 and AFP20; C_miR+AFP400_, the combination of miR-4651 and AFP400.

**Figure 4:**
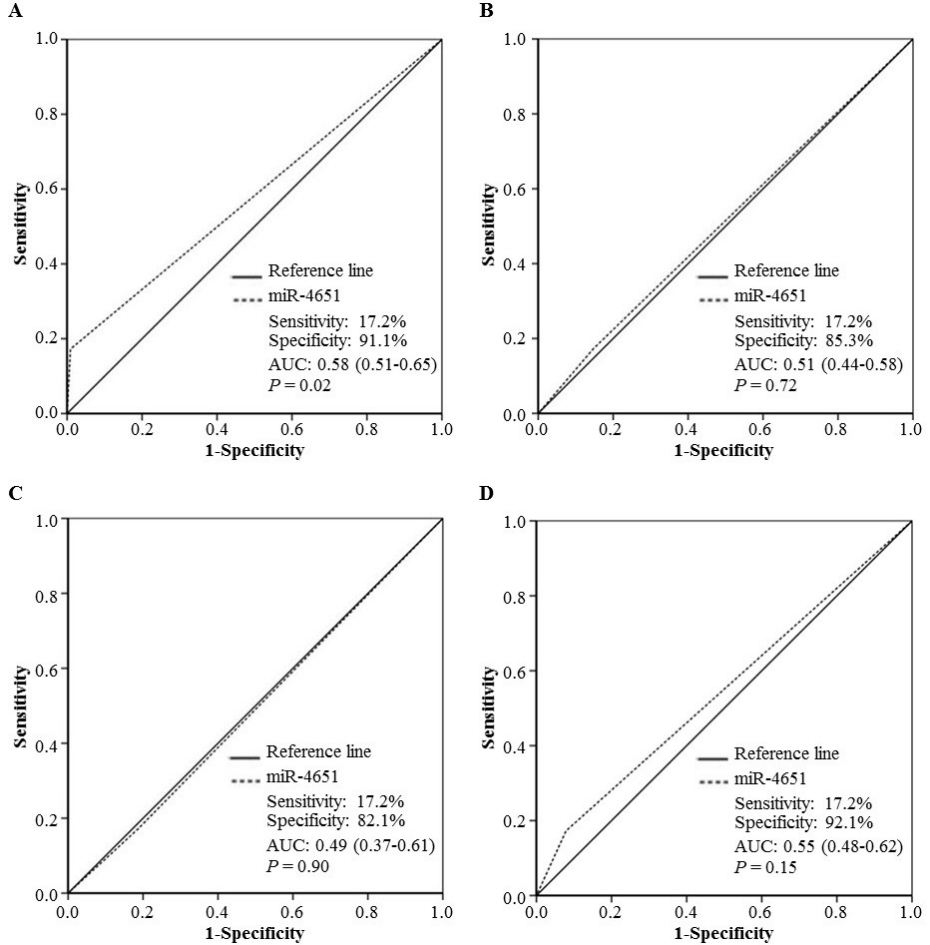
The diagnostic potential of miR-4651 for AFB1-negative hepatocellular carcinoma (HCC) The receiver operating characteristic curve (ROC) was used to differentiate patients with AFB1-negative HCC (Group 5, *n* = 87) from healthy controls without positive AFB1 exposure (Group 1, *n* = 338) **A.**, from healthy controls with positive AFB1 (Group 2, *n* = 292) **B.**, from control with liver diseases exception tumors (Group 3, *n* = 32) **C.**, or the combination of Group 1, 2, and 3 (*n* = 662) **D.** *Abbreviations*: AUC, the area under the ROC curve.

According to the results presented above, we chose miR-4651 to further investigate whether serum levels could differentiate cases with AFB1-positive HCC from controls by the receiver operating characteristic (ROC) curves. Results showed that miR-4651 had the comparable area under ROC curves (AUC, 0.88; 95% confidence interval [CI], 0.86 - 0.91; *P* < 0.001), sensitivity (77.4%) and specificity (92.7%) to differentiate AFB1-positive HCC cases from controls (Figure 2). At the best cut-off value (9.306), miR-4651 had an AUC 0.85 (0.82-0.88) to discriminate cases with AFB1-positive HCC from controls with a sensitivity of 78.1% and specificity of 92.1% (Table 4 and Figure 3). However, miR-4651 in this setting did not detect AFB1-negative HCC cases from controls (*P* = 0.15, Figure 4). The diagnostic potential of serum miR-4651 and AFP for AFB1-positive HCC was further compared. The AUC for miR-4651 was not significantly different from AFP20, but was significantly greater than AFP400 (difference between areas = 0.13, *P* < 0.001) (Table 4 and Figure 3).

**Table 4:** Comparison of ROC curves for serum miR-4651 and AFP

	AUC (95% CI)	Sensitivity (%)	Specificity (%)	*p*-value*
Group 4 vs Group 1				
miR-4651	0.89 (0.86-0.92)	78.1	99.1	-
AFP20	0.81 (0.77-0.84)	61.3	100.0	<0.001/<0.001/0.25
AFP400	0.72 (0.67-0.76)	43.0	100.0	<0.001/<0.001/0.25
miR-4651 +AFP20	0.91 (0.88-0.93)	81.0	100.0	0.35/0.40/0.25
miR-4651 +AFP400	0.92 (0.89-0.94)	83.2	100.0	0.14/0.13/0.25
Group 4 vs Group 2				
miR-4651	0.82 (0.78-0.85)	78.1	85.3	-
AFP20	0.81 (0.77-0.84)	61.3	100.0	0.68/<0.001/<0.001
AFP400	0.72 (0.67-0.76)	43.0	100.0	<0.001/<0.001/<0.001
miR-4651 +AFP20	0.85 (0.81-0.88)	81.0	88.4	0.24/0.40/0.27
miR-4651 +AFP400	0.89 (0.86-0.92)	83.2	94.5	0.003/0.13/<0.001
Group 4 vs Group 3				
miR-4651	0.80 (0.71-0.88)	781.	81.2	-
AFP20	0.68 (0.59-0.78)	61.3	75.0	0.07/<0.001/0.55
AFP400	0.72 (0.65-0.90)	43.0	100.0	0.13/2.08×10^−17^/0.02
miR-4651 +AFP20	0.83 (0.75-0.90)	81.0	84.4	0.60/0.40/0.74
miR-4651 +AFP400	0.87 (0.81-0.93)	83.2	90.6	0.18/0.13/0.47
Group 4 vs Group 1+2+3				
miR-4651	0.85 (0.82-0.88)	78.1	92.1	-
AFP20	0.80 (0.76-0.84)	61.3	98.8	0.06/<0.001/<0.001
AFP400	0.72 (0.67-0.77)	43.0	100.0	<0.001/<0.001/<0.001
miR-4651 +AFP20	0.88 (0.85-0.91)	81.0	94.1	0.25/0.40/0.16
miR-4651 +AFP400	0.90 (0.87-0.93)	83.2	97.1	0.02/0.13/<0.001

*The *p* value indicates the statistical significance for the differences of AUC/sensitivity/specificity as compared with miR-4651. Abbreviations: ROC, the receiver-operating characteristic; AFP, α-fetoprotein; AUC, the area under the ROC curve; AFP20, the cutoff of 20 ng/mL AFP; AFP400, the cutoff of 400 ng/mL AFP; CmiR+AFP20, the combination of miR-4651 and AFP20; CmiR+AFP400, the combination of miR-4651 and AFP400.

**Figure 5:**
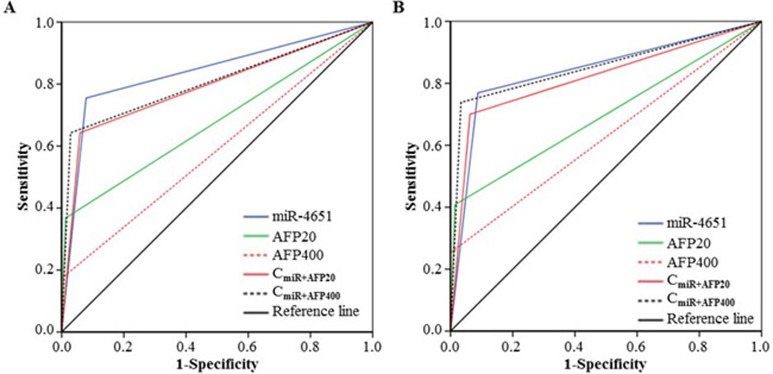
Performance to detect aflatoxin B1 (AFB1)-positive small and early-stage liver cancer The receiver operating characteristic curve was used to distinguish cases with AFB1-positive small (*n* = 98) **A.** or early-stage (*n* = 111) **B.** liver cancer from controls (*n* = 662). *Abbreviations*: AFP20, the cutoff of 20 ng/mL AFP; AFP400, the cutoff of 400 ng/mL AFP; C_miR+AFP20_, the combination of miR-4651 and AFP20; C_miR+AFP400_, the combination of miR-4651 and AFP400.

Compared with AFP20 and AFP400, miR-4651 showed larger AUC and higher sensitivity for both small-size (tumor size ≤ 3 cm) and early-stage HCC with positive AFB1 (Table 5 and 6, Figure 5). For example, miR-4651 was more sensitive than AFP20 (75.5% *vs.* 36.7%, *P* < 0.001) or AFP400 (75.5% *vs.* 17.3%, *P* < 0.001) with respect to identifying small-size HCC with positive AFB1. However, AFP20 and AFP400 had higher specificity to identify small-size tumors and early stage tumors (98.8%, 100.0%, and 92.1% for AFP20, AFP400, and miR-4651, respectively).

**Table 5:** Comparison of ROC curves for serum miR-4651 and AFP based on small liver cancer with positive aflatoxin B1

Variable	AUC (95% CI)	Sensitivity (%)	Specificity (%)	*p*-value*
miR-4651	0.84 (0.79-0.89)	75.5	92.1	-
AFP20	0.68 (0.61-0.75)	36.7	98.8	<0.001/<0.001/<0.001
AFP400	0.59 (0.52-0.65)	17.3	100.0	<0.001/<0.001/<0.001
miR-4651 +AFP20	0.79 (0.73-0.85)	64.3	94.1	0.25/0.09/0.16
miR-4651 +AFP400	0.81 (0.75-0.87)	64.3	97.1	0.43/0.09/<0.001

*The *p* value indicates the statistical significance for the differences of AUC/sensitivity/specificity as compared with miR-4651. Abbreviations: ROC, the receiver operating characteristic curve; AFP, α-fetoprotein; AUC, the area under the ROC curve; AFP20, the cutoff of 20 ng/mL AFP; AFP400, the cutoff of 400 ng/mL AFP.

**Table 6:** Comparison of ROC curves for serum miR-4651 and AFP based on the early HCC with positive AFB1

Variable	AUC (95% CI)	Sensitivity (%)	Specificity (%)	*p*-value*
miR-4651	0.84 (0.79-0.89)	75.7	92.1	-
AFP20	0.69 (0.63-0.75)	38.7	98.8	<0.001/<0.001/<0.001
AFP400	0.63 (0.56-0.69)	25.2	100.0	<0.001/<0.001/<0.00110^−13^
miR-4651 +AFP20	0.80 (0.75-0.89)	65.8	94.1	0.29/0.11/0.16
miR-4651 +AFP400	0.84 (0.79-0.89)	71.2	97.1	0.93/0.45/<0.001

*The *p* value indicates the statistical significance for the differences of AUC/sensitivity/specificity as compared with miR-4651. Abbreviations: ROC, the receiver operating characteristic curve; AFP, α-fetoprotein; AUC, the area under the ROC curve; AFP20, the cutoff of 20 ng/mL AFP; AFP400, the cutoff of 400 ng/mL AFP.

**Figure 6:**
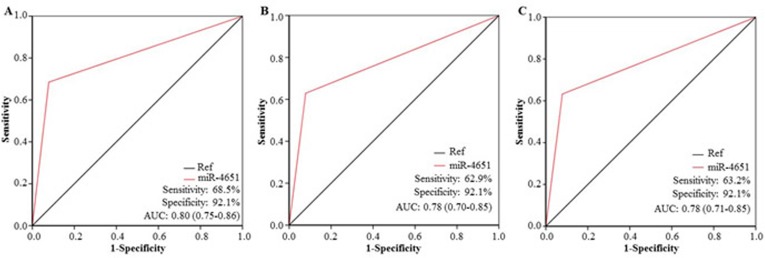
Performance to detect α-fetoprotein (AFP)-negative hepatocellular carcinoma (HCC) with aflatoxin B1 (AFB1) **A.** The receiver operating characteristic curve (ROC) was used to distinguish cases with both AFP-negative HCC and positive AFB1 (*n* = 108) from controls (*n* = 662). **B.** ROC was used to distinguish cases with both AFP-negative small liver cancer and positive AFB1 (*n* = 62) from controls (*n* = 662). **C.** ROC was used to distinguish cases with both AFP-negative early-stage HCC and positive AFB1 (*n* = 68) from controls (*n* = 662). *Abbreviations*: AUC, the area under the ROC curve; Ref, reference line.

Next, we analyzed the diagnostic performance of miR-4651 for both AFB1-positive and AFP-negative HCC (Figure 6). In this study, 38.7% (108/279) of individuals with AFB1-positive HCC were AFP-negative. The miR-4651 demonstrated high accuracy in discriminating individuals with AFB1-positive HCC but AFP-negative from controls (AUC, 0.80; 95% CI, 0.75-0.86) (Figure 6A). This microRNA had about 70% sensitivity and 90% specificity for detection of AFP-negative HCC. In accordance with the above-mentioned results, miR-4651 also exhibited value in identifying the AFP-negative HCC with a small size (Figure 6B) or at an early stage (Figure 6C).

### Serum miR-4651 significantly correlated with HCC prognosis

**Figure 7:**
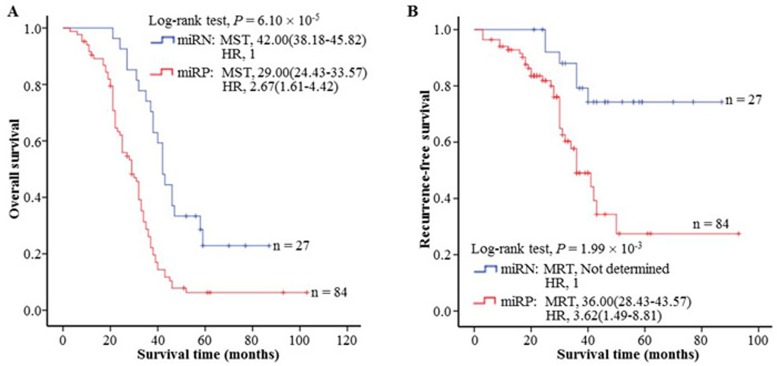
The association between the serum miR-4651 and HCC prognosis in 111 aflatoxin B1-positive cases with hepatocellular carcinoma (HCC) The serum levels of miR-4651 were found to correlate with the overall survival **A.** or tumor recurrence-free survival **B.** of HCC. Cumulative hazard function was plotted by Kaplan-Meier's methodology, and *P* value was calculated with two-sided log-rank tests. *Abbreviations*: MST, the median overall survival time; MRT, the median tumor recurrence-free survival time; miRN, the miR-4651-negative status; miRP, the miR-4651-positive status; HR, hazard ratio.

To study the effects of the serum miR-4651 on outcome of HCC patients, we analyzed the survival follow-up information of all HCC patients. Among these subjects, 111 received the same curative resection as the initial treatment but not did other treatments before resection, according to Chinese Manage Criteria of HCC [21], and were included for final survival analysis. We first analyzed the distribution difference of the miR-4651 status among different clinic-pathological characteristics of cases (Table 7). A significant difference between different serum levels of miR-4651 among AFP (odds ratio [OR] = 11.72, 95% CI = 2.59-53.01), but not in age, gender, race, tumor sized, tumor grade, or cirrhosis was observed. Results of the Kaplan-Meier survival analysis showed that increasing serum miR-4651 level significantly correlated with shorter overall survival (OS) and recurrence-free survival (RFS) of HCC cases (Figure 7A and 7B). From Cox regression analysis (Figure 7A and 7B) we showed that the miR-4651 is correlated with poor prognosis of HCC (miR-4651-positive risk value, hazard ratio [HR] = 2.67 and *P* = 1.44 × 10^−4^ for OS and 3.62 and 4.52 × 10^−3^ for RFS, respectively). Taken together, these results indicated that the serum miR-4651 is independent of other clinical covariates and suggested its potential as an independent prognostic factor for HCC.

**Table 7:** The serum miR-4651 and the clinic-pathological features of HCC cases with resection treatment

	miR-4651 negative (*n* = 27)		miR-4651 positive (*n* = 84)			
Features	*n*	%	*n*	%	OR (95% CI)	*P*
Age (years)						
≤ 48	16	59.3	42	50.0	Reference	
> 48	11	40.7	42	50.0	1.40 (0.58-3.39)	0.46
Gender						
Female	3	11.1	15	17.9	Reference	
male	24	88.9	69	82.1	0.61 (0.16-2.30)	0.46
Race						
Han	11	40.7	38	45.2	Reference	
Zhuang	16	59.3	46	54.8	0.85 (0.35-2.06)	0.72
Tumor size						
≤ 3 cm	20	74.1	56	66.7	Reference	
> 3 cm	7	25.9	28	33.3	1.43 (0.54-3.78)	0.48
Edmondson differentiation grade						
I-II	15	55.6	35	41.7	Reference	
III-IV	12	44.4	49	58.3	1.67 (0.69-4.05)	0.26
Liver cirrhosis						
No	19	69.2	62	73.8	Reference	
Yes	8	30.8	22	26.2	0.80 (0.30-3.11)	0.66
AFP20						
Negative	25	92.6	43	51.2	Reference	
Positive	2	7.4	41	48.8	11.72 (2.59-53.01)	1.40×10^−3^
AFP400						
Negative	26	96.3	57	67.9	Reference	
Positive	1	3.7	27	32.1	11.97 (1.53-93.74)	0.02

Abbreviations: HCC, hepatocellular carcinoma, AFB1, aflatoxin B1.

### Serum miR-4651 positively associated with serum AAA levels

**Figure 8:**
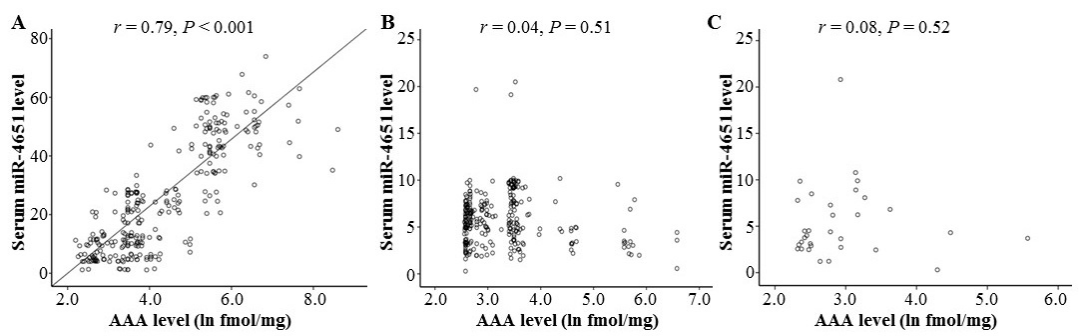
The correlation between the serum aflatoxin B1 (AFB1)-albumin adduct (AAA) levels and miR-4651 levels among individuals with positive AFB1 exposure AAA and miR-4651 were tested using the comparative enzyme-linked immunosorbent assay and TaqMan-PCR techniques, respectively. The serum levels of miR-4651 were positively associated with the serum AAA levels among HCC cases with positive AFB1 exposure **A.**, but not among healthy controls with positive AFB1 exposure **B.** or controls with both positive AFB1 exposure and liver diseases exception tumors **C.**

To investigate whether AFB1 exposure was associated with miR-4651 expression, we explored the correlation between serum AAA levels and serum miR-4651 levels in subjects with positive AFB1. The increasing serum levels of miR-4651 were found among HCC cases with higher serum AAA levels (Figure 8A). Spearman's correlation analysis further exhibited the serum miR-4651 levels were positively related with the serum levels of AAA (*r* = 0.79, *P* < 0.01). However, this positive association between miR-4651 levels and AAA levels was not observed among controls with positive AFB1 (Figure 8B and 8C). Taken together, our data suggested that miR-4651 expression might be correlated with AFB1 exposure among cases with positive AFB1.

## DISCUSSION

Increasing studies have proved that microRNAs have a crucial role in human carcinogenesis, including hepatocarcinogenesis, via acting as oncogenes or tumor suppressor genes [22–25]. Recent evidence has shown that serum microRNAs are remarkably stable and expression patterns may be tissue-specific; thus, they may be important potential candidates for non-invasive cancer testing [26]. It has been hypothesized that serum microRNAs might have diagnostic potential for HCC, including AFB1-related HCC. Therefore, in the present we conducted a case-control study to screen and analyze potential serum microRNAs for the early diagnosis of AFB1-related HCC in a high AFB1 exposure area, Guangxi area of China [27]. Our results proved that serum microRNAs have different expression between HCCs and non-HCCs, and exhibit useful diagnostic value. Supporting our findings, recent several reports have shown that serum microRNAs could detect HCC cases from controls [2, 14–16, 18, 24, 28–33]. For example, Wang *et al.* [18] investigated the differential profiles of circulating microRNAs expression in HBV-related small liver cancer and found six differentially expressed serum microRNAs in HCC related to HBV. Yu *et al.* [16] and Li *et al.* [32] identified miR-150 and miR-18a as potential markers for hepatitis B virus-related HCC screening. Motawi *et al.* [14] and Lin *et al*. [31] analyzed the combination value of several serum microRNAs in the early-stage diagnosis of HCC, and found microRNA classifier should be more valuable to detect HCC. Collectively, these results suggest that serum microRNAs might be important biomarkers for HCC diagnosis.

In this study, miR-4651 was particularly concerned because of its different expression between HCC patients with and without positive-AFB1 status. This microRNA, a kind of small RNA encoded by *miR-4651* gene (located at chr7: 75915197-75915269), was found in breast cancer [34]. Until now, it has been not clear whether miR-4651 acts as a tumor suppressor or an oncogene. However, we observed that miR-4651 had higher expression in the AFB1-positive HCC cases than in non-HCC-harboring individuals, and this increasing expression was further positively associated with AAA levels among AFB1-positive HCCs and poor prognosis of HCC related to AFB1. This implies that miR-4651 might act as an oncogene or have a similar role of oncogenes through decreasing detoxication and genomic DNA damage repair capacity because the levels of AAA correlate with HCC risk and prognosis, and can reflect the deficiency of detoxication and genomic DNA damage repair capacity [35]. Interestingly, miR-4651 had higher accuracy and sensitivity to identify small liver cancer and early-stage AFB1-positive HCC compared with AFP; highlighting its diagnostic value in AFP-negative tumor cases. Taken together, these results implied that miR-4651 might be a useful diagnostic biomarker for HCC induced by AFB1 exposure.

This study has several strengths. First, we completed a high-throughput screening analysis for serum microRNAs that exhibited differential levels between HCC cases with positive-AFB1 status and healthy controls featuring risk factor AFB1 exposure. Through this methodology, we not only improved the chance to identify serum biomarkers, but obtained 8 possible AFB1-related microRNAs. Second, to control effects of other carcinogenetic factors such as HBV and HCV, only HBV- and HCV-negative cases were included in this study; whereas HBV- or HCV-positive individuals were excluded. Third, the present study included both healthy controls without risk factor and controls with different risk stages, where the serum levels of microRNAs could change at the different states. Finally, a control group whose patients with HCC were not caused by AFB1 exposure was included in the present study to evaluate whether miR-4651 had the similar diagnostic potential for non-AFB1-related HCC. Our data showed that miR-4651 can distinguish individuals with AFB1-positive HCC from controls with different risk stages. However, this microRNA did not exhibit similar diagnostic value for AFB1-negative cases. This implies the practicality of miR-4651 in clinical practice.

However, there were several limitations to our study. First, potential selection bias might have occurred through the selection of hospital-based control subjects. Second, although the serum levels of miR-4651 among AFB1-positive HCC cases were significantly associated with serum AAA levels, we did not investigate the association between miR-4651 and other known causes of HCC such as HBV and HCV. Finally, we did not examine additional functional analysis. Thus, more functional analyses should be performed based on large samples and a combination of biomarkers and different causes.

In summary, this study represents the first report describing the serum levels of microRNAs and their early diagnostic and prognostic roles in AFB1-related HCC. The findings provide an additional insight into serum miR-4651 as a biological determinant for identifying small-size, early-stage, and AFP-negative HCC related to AFB1. Therefore, these findings, in combination with AFP, could improve the identification of HCC, especially in high AFB1-exposure areas.

## MATERIALS AND METHODS

### Study design and participants

We conducted a hospital-based case-control study of AFB1-related HCC in the Guangxi area. To validate biomarkers, the present study consists of three control groups and two case groups (Table 1): healthy controls without positive AFB1 (Group 1), healthy controls with positive AFB1 (Group 2), controls with positive AFB1 and liver diseases exception tumors (Group 3), patients with positive AFB1 and diagnosed HCC (Group 4), and cases with diagnosed HCC but without positive AFB1 (Group 5). All newly diagnosed HCC patients in hospitals affiliated with Youjiang Medical College for Nationalities and Guangxi Medical University from January 2006 to December 2010 were utilized. All controls were recruited from the general health check-up center at the same hospitals during the same period for comparison. To control the effects of confounders, controls were individually matched to controls based on gender, ethnicity (Han, Zhuang), age (± 5 years), time when sampled, and hospital locations. All controls were surveyed to ascertain their willingness to participate in the study and to provide preliminary demographic data. In this study, a total of 366 cases and 662 controls, representing 92% of eligible cases and 95% of eligible controls were interviewed and included the final analysis.

In this report, all participants were defined as Group 1 (*n* = 338), 2 (*n* = 292), 3 (*n* = 32), 4 (*n* = 279), or 5 (*n* = 87) by medical doctors, according to eligibility criteria listed in Table 1. All HCC cases (including AFB1-negative and -positive HCC cases) were confirmed histopathologically according to the American Association for the Study of Liver Diseases (AASLD) guidelines. Tumour stage was defined according to the Barcelona Clinic Liver Cancer (BCLC) staging system, and tumours with BCLC stage 0 and stage A were defined as early-stage HCC. Liver cirrhosis was evaluated by biopsy or hepatic ultrasound technology with CT or MRI. All controls underwent serial monitoring of AFP, ultrasonography, and hepatitis virus biomarkers, and there was not any evidence of HBV, HCV, or tumors. The controls were screened at baseline; whereas cases were screened at primary diagnostic time. Written informed consent was obtained for all participants and 5 mL of peripheral while blood was collected along with demographic data. This study was approved by the Institutional Ethics Committee of Youjiang Medical University for Nationalities, and was carried out in accordance with the approved guidelines (No. AYJM20090112).

### Serum preparation

For serum preparation, 5 mL peripheral whole blood was collected into drying tubes, and then was centrifuged at 4°C, 3000 r.p.m. for 10 min, which was followed by an additional centrifugation at 12,000 r.p.m. for 15 min to completely remove all remaining cells. The serum samples were aliquoted and stored at -80°C until analysis.

### Laboratory tests

Fasting venous blood samples were collected from all patients for routine workup, including complete blood picture, liver function tests, prothrombin concentration and prothrombin international normalized ratio, AFP, and HBV and HCV markers using commercially available assays. In this report, AFP20 and AFP400 were defined as the cutoffs of 20 ng/mL and 400 ng/mL AFP, respectively.

### AFB1 status analysis

Because the serum AAA is a stable AFB1 exposure biomarker [36], the levels of AAA were used to evaluate AFB1 exposure levels of all subjects. AAA levels in the serum were tested using the comparative enzyme-linked immunosorbent assay as previously published [37]. According to our previous reports with respect to AFB1 exposure [37], value of > 2.18 ln fmol/mg was considered as positive-AFB1 status.

### Serum microRNAs expression profiling analysis

In this report, we screened and optimized the serum microRNAs *via* two methods: RT^2^ Profiler PCR Array and TaqMan-PCR analysis. For the PCR Array analysis, we randomly selected six HCC cases with positive AFB1 from Group 4 and six age-, sex-, race-, and sample collected time and location-matched controls with positive AFB1 exposure but without any evidence of liver tumors from Group 2. The sera were collected from these individuals and sent to Shanghai Oe-Bio-Tech Medical Company (Shanghai, China) for microRNA detection. Briefly, total RNA from 1 mL serum was extracted with the PAXgene^®^ Blood RNA Kit (cat#762174, Qiagen, Dusseldorf, Germany). RNA concentration and purity were evaluated on a NanoDrop spectrophotometer and RNA integrity (RIN) was evaluated on a BioAnalyzer 2100; the results showed good RNA quality (RIN value 7.2). The relative cDNA was synthesized by RT^2^ First Strand Kit (cat#330401, Qiagen). After that, the amounts of human microRNAs in the serum samples were tested *via* the real-time PCR (on an Applied Biosystems 7900HT Real-Time PCR System) using RT^2^ Profiler PCR Arrays (cat# PAHS-028ZF-2, Qiagen) in combination with RT^2^ SYBR Green Mastermixes (cat# 330500, Qiagen). The cycle threshold (CT) values were processed and subsequently analyzed using the ^ΔΔ^CT method by the PCR Array Data Analysis Web portal (www.SABiosciences.com/pcrarraydataanalysis.php). Briefly, the relative expression level of candidate microRNAs to endogenous control cel-miR-39-3p was calculated as 2^−ΔCT^, where ΔCT = CT_microRNA_ - CT_cel-miR-39-3p_. To determine fold change in gene expression, the normalized expression of the candidate microRNAs in the case group is divided by the normalized expression of the same microRNAs in the control group, also called 2^−ΔΔCT^, where ΔΔCT = CT_(case group)_ - CT_(control group)_. In this phase, a total of 8 candidate microRNAs were chosen for TaqMan-PCR analysis in all participants according to the following criteria: >2-fold change between AFB1-positive HCC cases and controls with positive AFB1, and coefficient of variation (CV) CT < 0.05, and high expression (CT_average_ < 29 cycles) in AFB1-positive HCCs.

### TaqMan-PCR assay for candidate microRNAs in the training and validation study

The serum levels of 8 candidate microRNAs were tested using quantitative reverse transcription-PCR with TaqMan probe described in our previous reports [5, 6]. Briefly, total RNA was extracted from 400 μL serum with 0.2 nM of cel-miR-67 using PureLink^®^ RNA Mini Kit (cat#12183018A, Ambion, USA), and corresponding first-strand cDNAs were synthesized using High Capacity cDNA Reverse Transcription Kit (cat# 4368814, Invitrogen Grand Island, NY) and TaqMan MicroRNA Reverse Transcription Kit (cat#4366596, Applied Biosystems, Carlsbad, CA). After that, TaqMan-PCR analysis was performed using standard protocols on a Bio-Rad iCycler CFX Detection System. The serum levels of candidate microRNAs were assessed using TaqMan microRNA assays (cat#4427975, Applied Biosystems) with cel-miR-67 as the endogenous control. PCR reactions were run in a 5-μL final volume containing 1 TaqMAN Universal Master Mix II (cat#4440041, Applied Biosystems), 1 × TaqMan microRNA probes and primers (cat#4427975, Applied Biosystems), and about 15 ng of cDNA. Cycling conditions were 95°C for 30 s, and 50 cycles of 95 C for 15 s and 60 C for 1 min. All reactions were conducted in triplicate and controls were performed with no template or no reverse transcription for each gene. In this study, the relative amount of candidate microRNAs to cel-miR-67 was calculated as 2^−ΔCT^ method, where ΔCT = CT_microRNA_ - CT_cel-miR-67_. The microRNAs with significantly different expression levels between cases and controls were further selected for performance analysis in the training and validation study. To avoid bias, RNA extraction and PCR analysis for all samples were performed by two different investigators (W.-X.M. and H.-H.D.) without knowledge of the case-control status. Each PCR analysis was accomplished with the controls as positive, negative, and blank controls, respectively. When any of these controls failed, PCR was re-conducted.

### HCC following-up

Patients with HCC were followed and underwent serial monitoring of AFP, ultrasonography, chest radiograph, and emission computed tomography every 2 months for the first 2 years and semiannually thereafter for detection of recurrence. Recurrence was confirmed by imaging techniques, either intrahepatically or extrahepatically (lymph nodes, distant metastases). An increase of AFP without radiologic evidence of a new tumor was not regarded as recurrence until confirmed by imaging. The last follow-up day was set on 31 December 2015, and the survival status was confirmed by means of clinic records and patient or family contact. We defined the duration of OS as from the date of curative treatment to the date of death or last known date alive. The duration of RFS was defined as the date of curative treatment to the date of tumor recurrence or last known date alive.

### Statistical analysis

All statistical analyses were performed using the SPSS version 18 (SPSS Institute, Chicago, IL). The differences of age, race, gender, and liver function between groups were compared using Student *t* test and the χ^2^ test. The non-parametric Mann-Whitney *U*-test was used for comparison of microRNA data (2^−ΔCT^) from independent samples from 2 groups as this type data were not normally distributed. The diagnostic value of miR-4651, AFP20, and AFP400 for HCC was analyzed using ROC curve. The difference of AUC was used as an accuracy index for evaluating the diagnostic performance. Spearman's correlation analysis was used to elucidate the association between the serum AAA levels and miR-4651 levels. Kaplan-Meier survival analysis with the log-rank test was used to evaluate the effects of the serum miR-4651 levels on HCC prognosis. Risk factors for HCC prognosis were selected using the Cox multivariate regression model with stepwise forward selection based on a likelihood ratio test. HRs and 95% CIs for risk factors were then calculated from a multivariate Cox regression model. All statistical tests were 2-tailed, and a *P*-value of less than 0.05 was considered statistically significant.

## Abbreviations

HCC: hepatocellular carcinoma
AFB1: aflatoxin B1
AFP: α-fetoprotein
AFP20: the cutoff of 20 ng/mL AFP
AFP400: the cutoff of 400 ng/mL AFP
C_miR+AFP20_: the combination of miR-4651 and AFP20
C_miR+AFP400_: the combination of miR-4651 and AFP400
miR-4651: microRNA-4651
HBV: hepatitis virus B
HCV: hepatitis virus C
AAA: AFB1-albumin adducts
ROC: receiver operating characteristic
AUC: area under the ROC curve
AST: aspartate aminotransferase
ALT: alanine aminotransferase
